# Similarity of inflammatory response in epileptic seizures and sepsis: does the sensitivity to sepsis in epileptic patients increase?

**DOI:** 10.1186/cc14059

**Published:** 2014-12-03

**Authors:** G Üzüm, K Akgün-Dar, A Kandil, N Bahçekapılı, I Albeniz

**Affiliations:** 1 Department of Physiology, Medical Faculty of Istanbul, Istanbul University, Istanbul, Turkey; 2 Department of Biology, Faculty of Science, Istanbul University, Istanbul, Turkey

## Introduction

It is known that the systemic response during sepsis is caused by proinflammatory mediators such as interleukin-1 (IL-1), interleukin-6 (IL-6) and tumor necrosis factor alpha (TNFα). The inflammatory response in sepsis causes disorders in the brain in addition to multiorgan dysfunctions including the kidneys and liver [1]. Inducible nitric oxide synthase (iNOS) is induced in hepatocytes by sepsis and mediates hepatic injury [2]. Matrix metalloproteinases (MMPs) play an important role in the formation of sepsis and mediate inflammatory response and tissue damage [3]. On the other hand, there are some cases in which systemic inflammatory response occurs without the presence of infection such as epilepsy. Cytokines are well-known inflammatory mediators in the brain, and they increase following seizures. We previously demonstrated that pentyleneterazol (PTZ)-induced generalized epileptic seizures significantly increased inflammatory markers (TNFα, IL-1β, IL-6) in the brain and S100B in serum [4]. In this preliminary study, we aimed to investigate the MMP2, MMP9, NOS, and myeloperoxidase activity in the liver and kidney and levels of serum proinflammatory cytokines following PTZ-induced generalized clonic-tonic seizures.

## Methods

Adult Sprague-Dawley rats were divided into two groups as Control and PTZ groups. The Control group was given saline and the PTZ group was given 80 mg/kg PTZ i.p. Two hours after seizures, the rats were decapitated and a cardiac blood sample was drawn, and liver and kidneys were removed. Proinflammatory markers (IL-1βN, TNF-α, IL-6) were investigated in serum by ELISA. eNOS, iNOS, MMP2, and MMP9 levels were analyzed immunohistochemically in the liver and kidney.

## Results

**Figure 1:**
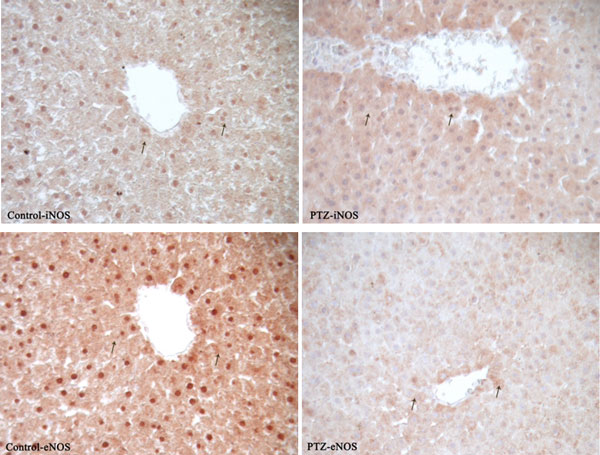
**Immunohistochemical detection of iNOS and eNOS staining (arrows) in liver sections in control and experimental groups (bar: 50 μm)**.

**Figure 2:**
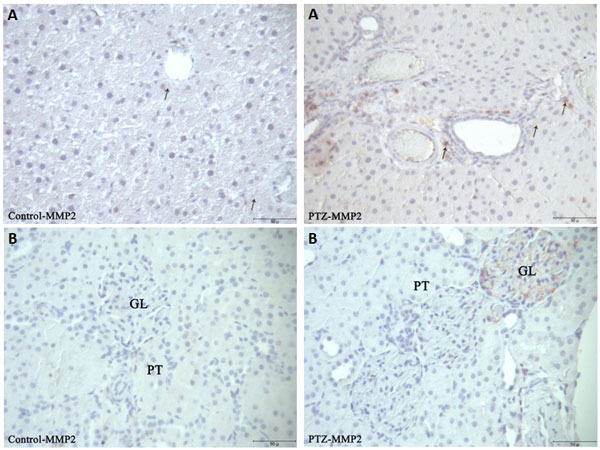
**Immunohistochemical detection of MMP2 staining (arrows) in liver **(A) **and kidney **(B) **sections in control and experimental groups**. PT, proximal tubule; GL, glomerulus (bar: 50 μm).

**Figure 3:**
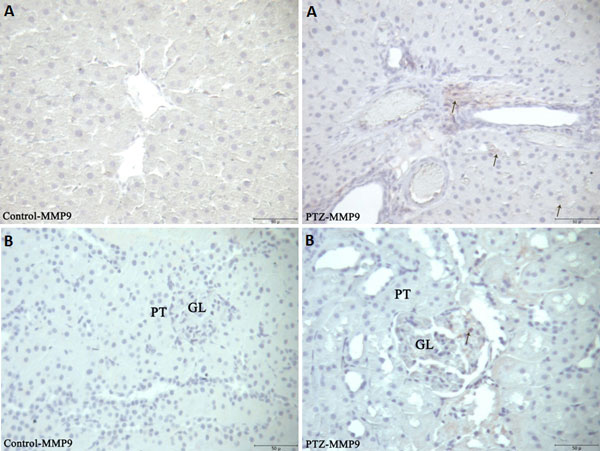
**Immunohistochemical detection of MMP9 staining (arrows) in liver **(A) **and kidney **(B) **sections in control and experimental groups**. PT, proximal tubule; GL, glomerulus (bar: 50 μm).

**Figure 4:**
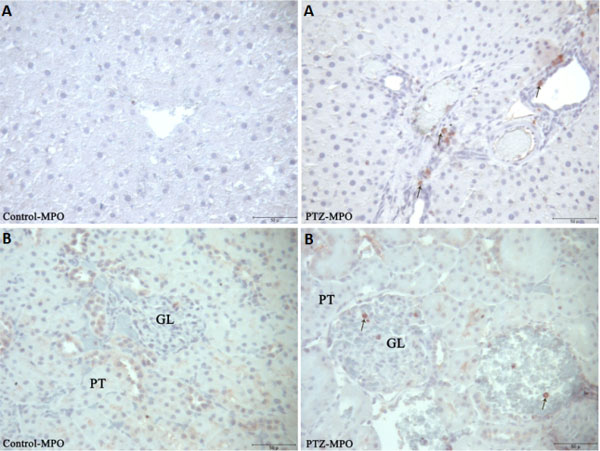
**Immunohistochemical detection of MPO staining (arrows) in liver **(A) **and kidney **(B) **sections in control and experimental groups**. PT, proximal tubule; GL, glomerulus (bar: 50 μm).

Proinflammatory markers significantly increased in the serum of rats after PTZ-induced seizures (Table 1). iNOS reaction was markedly increased while eNOS reactions were decreased (Figure 1) in the liver of rats after PTZ-induced seizures. MMP2 in the central vein of the liver and connective tissue areas of liver and kidney tissues in the PTZ group were markedly increased (Figure 2). MMP9 immune reaction in the PTZ group slightly increased in the kidney and liver (Figure 3). MPO reactions, which are an indicator of inflammatory activity, were markedly increased in both tissues (Figure 4).

**Table 1:** Proinflammatory cytokines in control versus experimental groups

pg/ml	Control	PTZ
IL-1β	0.117 ± 0.042	0.775 ± 0.064
TNFα	0.062 ± 0.010	0.783 ± 0.044
IL-6	0.160 ± 0.012	0.0654 ± 0.026

*P < 0.001, compared to control one-way ANOVA and Benferroni for *post hoc *were used. *n *= 7.

## Conclusion

The first findings show that long-term generalized clonic-tonic seizures markedly increase markers that mediate inflammation (iNOS, especially MMP2, MMP9, MPO) in the liver and kidney such as sepsis. In addition, proinflammatory markers (TNFα, IL-1β, IL-6) were found significantly high in serum. Thus, it is concluded that it will be worthwhile to determine whether epileptic seizures cause sensitivity to sepsis.
